# Thrombotic Storm With Budd-Chiari Syndrome in a Pediatric Patient With Ulcerative Colitis

**DOI:** 10.14309/crj.0000000000000159

**Published:** 2019-10-25

**Authors:** Rohit Josyabhatla, Diane Hsu, Michael McGuire, Sharon D'Mello

**Affiliations:** 1 Department of Pediatrics, Rutgers NJMS, Newark, NJ; 2 Division of Pediatric Radiology, Hackensack University Medical Center, Hackensack, NJ; 3 Division of Pediatric Gastroenterology, Hackensack University Medical Center, Hackensack, NJ

## Abstract

Ulcerative colitis is associated with an increased risk of thromboembolic phenomena. Thrombotic storm defined by the development of multiple thrombi in multiple locations within a short period of time is a rare condition that is potentially life threatening. We present a 14-year-old adolescent boy with an ulcerative colitis flare complicated by Budd-Chiari syndrome and thrombotic storm.

## INTRODUCTION

Ulcerative colitis (UC) is a type of chronic inflammatory bowel disease (IBD). Multiple genetic and environmental factors have been implicated as probable triggers.^1^ IBD often predisposes patients to a procoagulant state, leading to thromboembolic events (TEs).^2^ Multiple factors including thrombocytosis, upregulation of coagulation factors, downregulation of fibrinolytic factors, endothelial dysfunction, and the presence of procoagulant microvesicles have been suggested as possible explanations.^2^ Episodes of acute severe UC require hospitalization that is often accompanied by dehydration, immobilization, infection, and possible surgery, which further increase the risk of thrombosis.^3^ We present a 14-year-old adolescent boy with UC who presented with acute severe colitis and went on to develop Budd-Chiari syndrome (BCS) with thrombotic storm (TS).

## CASE REPORT

A 14-year-old adolescent boy with UC presented with a 2-week history of multiple episodes of bloody diarrhea, tenesmus, rectal pain, fever, fatigue, and 10-pound weight loss. He was diagnosed with UC 3 years before presentation, and his course so far had been uncomplicated while on treatment with sulfasalazine. He was admitted for management of his acute severe colitis. His hospital course was complicated by ongoing hematochezia, severe anemia (hemoglobin 8.3 gm/dL), and hypoalbuminemia (2.2 g/dL). Early in his course, he developed chest pain, shortness of breath, and hypotension refractory to fluids. Chest x-ray showed interstitial markings and small pleural effusions with a paucity of vascular markings. Electrocardiography showed ST depression in the inferior leads. Troponin (8.06 ng/mL) and brain natriuretic peptide (1,440 pg/mL) levels were elevated. Echocardiogram revealed mild left ventricular dilation with reduced ejection fraction of 40% with normal right ventricular size and function. The findings were attributed to demand ischemia. He was treated with blood transfusions and isotonic crystalloid infusions. Methylprednisone was initiated to better control the UC flare, and piperacillin/tazobactam was empirically added to cover for possible bacterial colitis. Colonoscopy was performed, and the colon biopsy showed ulcerated granulation tissue consistent with active colitis. The biopsy was negative for cytomegalovirus immunostaining.

**Figure 1.**
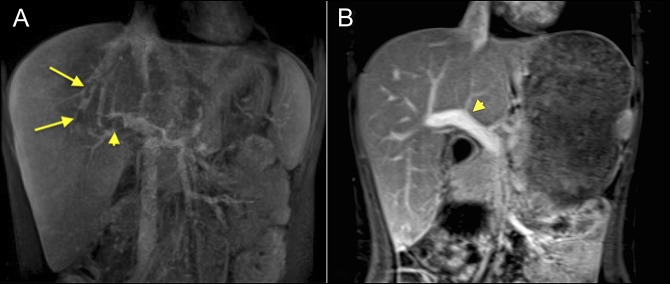
Coronal view of abdominal magnetic resonance imaging (MRI) (A) at admission showing filling defects within the right hepatic vein (arrows) and right portal vein (arrowhead) and (B) follow-up showing a near resolution of thrombus in the portal vein (arrowhead).

**Figure 2.**
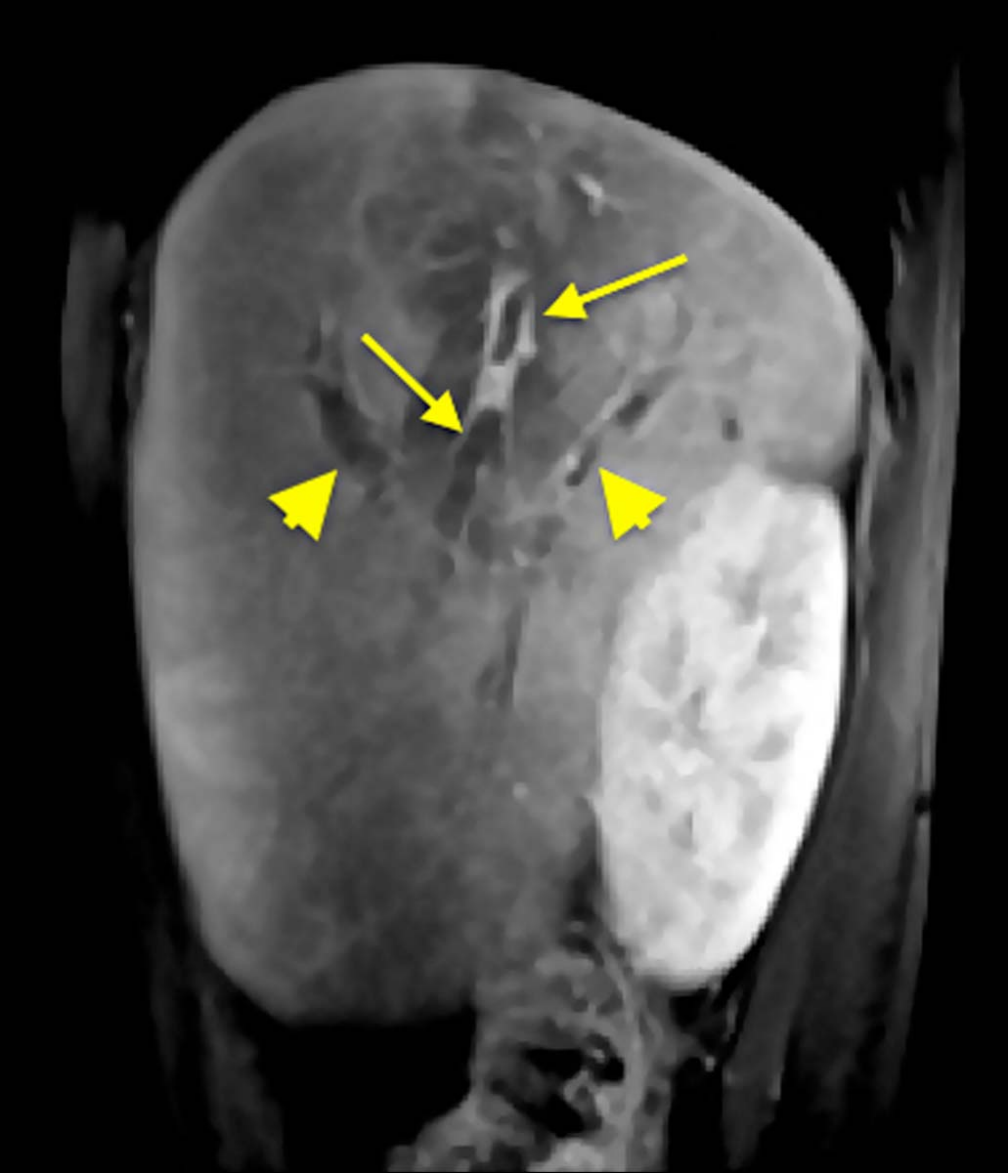
Sagittal view of abdominal MRI showing filling defects within the right hepatic vein (arrows) and right portal veins (arrowheads).

After starting methylprednisolone, his chest pain improved and the troponin and brain natriuretic peptide levels decreased, but he continued to have persistent bloody diarrhea, fever and leukocytosis (white blood cell count 12.9 × 10^3^/mcL). He then received 1 dose of infliximab but continued to remain symptomatic. Blood cultures, stool cultures, gastrointestinal pathogen panel, and *Clostridrium difficile* toxin assays were negative throughout. Computed tomography (CT) of the abdomen revealed diffuse hepatic parenchymal disease and focal attenuations in both kidneys, suggestive of renal infarcts (Figure 1). Magnetic resonance imaging (MRI) abdomen showed multiple nonocclusive thrombi in the portal venous system, thrombi in all 3 hepatic veins, and decreased enhancement in both kidneys, likely representative of renal infarcts (Figure 2). A diagnosis of TS including BCS was made, and he was started on heparin. Thrombophilia workup was negative.

**Figure 3.**
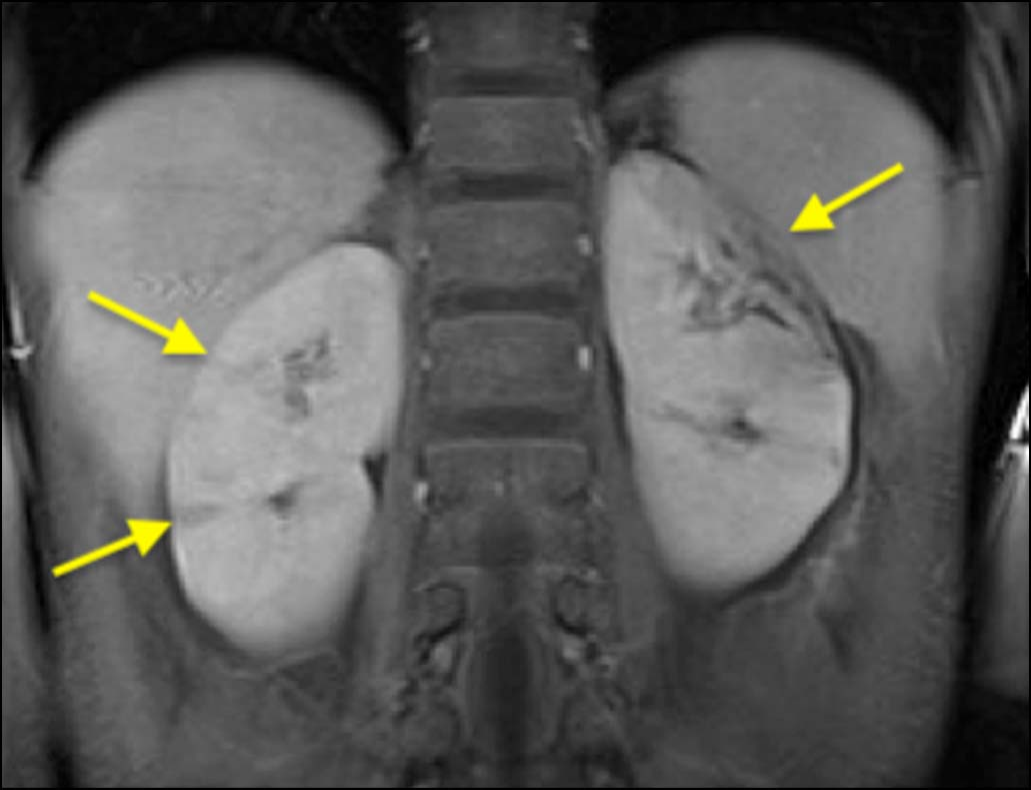
Coronal view of abdominal MRI with abnormal enhancement of the renal parenchyma bilaterally (arrows), with focal atrophic changes on the left.

Because of ongoing bloody diarrhea with steroid therapy and infliximab, and in light of a new diagnosis of TS with BCS, he underwent open subtotal colectomy with end ileostomy. The steroids were tapered, and he was discharged home on rectal hydrocortisone and enoxaparin for anticoagulation. Follow-up MRI 4 months after hospitalization showed a near complete resolution of the thrombi (Figure 3). Follow-up echocardiograms showed progressive improvement with subsequent normalization in the left ventricle function.

## DISCUSSION

A study by Nylund et al found that the relative risk for thromboembolism in hospitalized children and adolescents with IBD was 2.36 when compared with hospitalized children without IBD.^4^ One large retrospective analysis of a single hospital database found that thromboembolic complications occurred in 1.3% of the study population with UC.^5^ They reported that deep vein thrombosis (DVT) and pulmonary embolism were the most common culprits contributing to 60% of the cases, with a mortality rate as high as 25%. In pediatric IBD, a recent systematic review of the literature found that UC was more frequently associated with TE than with Crohn's disease.^6^ They also found that the most common sites for TE in pediatric IBD were cerebral (54.3%) and limbs (26%), while abdominal TE contributed to 13%. Only 6 pediatric cases of BCS with UC have been reported so far.^7–12^ What made our patient unique was the concurrent development of portal thrombi, hepatic venous thrombi, and renal infarcts.

TS, a rare entity in patients with a hypercoagulable state, is characterized by development of multiple thrombi in different sites in quick succession.^13^ Our patient had concurrent development of multiple renal infarcts and portal and hepatic venous thrombi, fitting the diagnosis of TS in the setting of fulminant colitis. Interestingly, the cardiac dysfunction, with the elevated brain natriuretic peptide and troponin levels, was attributed to demand ischemia, given the severe anemia with hypotension. However, in retrospect, the possibility of coronary arterial thrombi cannot be excluded. This makes it only the second presentation of a TS including BCS in UC. Maggi et al have previously described a 14-year-old girl who presented with BCS and developed cerebral vein thrombosis, right atrial thrombosis, and bilateral pulmonary embolism, despite anticoagulant therapy.^10^ However, in their patient, the TS was the first manifestation of a previously unknown UC.

Venous outflow obstruction of the hepatic venules, hepatic veins, the inferior vena cava, or the right atrium define the group of conditions that jointly comprise BCS.^14^ The lack of clinical signs in our patient made it a difficult diagnosis. Imaging plays an important role in the diagnosis of BCS. Doppler ultrasonography of the liver is the initial test of choice, while contrast-enhanced computed tomography or MRI is used to better identify the necrotic areas.^15–17^ Medical management strategies for BCS include (i) managing ascites with fluid, salt restriction, and use of diuretics, (ii) managing thrombi with anticoagulants, and (iii) treating the underlying causes.^18^ The options to overcome the hepatic outflow tract obstruction include thrombolytic therapy, angioplasty, and transjugular intrahepatic portosystemic shunt or surgical shunts.^19–21^ Liver transplant is reserved for patients with fulminant hepatic failure, cirrhosis, or shunt failure.^18^ BCS associated with UC has a poor prognosis, with rare cases of spontaneous regression.^22,23^

The management of BCS in UC is particularly challenging because of the fear of worsening hemorrhage with the use of anticoagulants. The management of TE in IBD has been heterogeneous in both adult and pediatric patients.^24^ The duration of anticoagulation remains variable and clinician dependent.^25^ Of the 7 pediatric patients with BCS and UC, 6 were treated with systemic anticoagulation. It is notable that none of these patients had bleeding complications.

There is an increased need for close surveillance of thromboembolic complications in the IBD patient population. At present, there are no formal guidelines for thromboprophylaxis in pediatric patients with IBD. Further studies are needed to understand the safety profile of anticoagulants in pediatric IBD and to establish clear guidelines on their choice, indications of use, and duration of therapy.

## DISCLOSURES

Author contributions: R. Josyabhatla wrote the manuscript. D. Hsu edited the manuscript. S. D'Mello revised the manuscript and is the article guarantor.

Financial disclosure: None to report.

Informed consent was obtained for this case report.
